# The application of zebrafish model in the study of cleft lip and palate development: A systematic review

**DOI:** 10.1016/j.heliyon.2024.e28322

**Published:** 2024-03-16

**Authors:** Nora Alhazmi, Khalid A. Abalkhail, Farraj Albalawi, Bassam Alalola, Fathima F. Farook

**Affiliations:** a Preventive Dental Science Department, College of Dentistry, King Saud bin Abdulaziz University for Health Sciences, Riyadh, 14611, Saudi Arabia; b King Abdullah International Medical Research Center, Riyadh, 11481, Saudi Arabia; c Ministry of the National Guard Health Affairs, Riyadh, 11426, Saudi Arabia

**Keywords:** Zebrafish, Danio rerio, Ethmoid plate, Cleft lip, Cleft palate, Cleft, *IRF6*

## Abstract

**Objective:**

Craniofacial growth and development are more than a scientific curiosity; it is of tremendous interest to clinicians. Insights into the genetic etiology of cleft lip and palate development are essential for improving diagnosis and treatment planning. The purpose of this systematic review was to utilize a zebrafish model to highlight the role of the *IRF6* gene in cleft lip and palate development in humans.

**Data:**

This review adhered to the guidelines outlined in the PRISMA statement. Nine studies were included in the analysis.

**Sources:**

This study used major scientific databases such as MEDLINE, EMBASE, Web of Science, and the Zebrafish Information Network and yielded 1275 articles. Two reviewers performed the screening using COVIDENCE™ independently, and a third reviewer resolved any conflicts.

**Study selection:**

After applying the inclusion and exclusion criteria and screening, nine studies were included in the analysis. The Systematic Review Center for Laboratory Animal Experimentation's (SYRCLE's) risk-of-bias tool was used to assess the quality of the included studies.

**Results:**

The main outcome supports the role of the *IRF6* gene in zebrafish periderm development and embryogenesis, and *IRF6* variations result in cleft lip and palate development. The overall SYRCLE risk of bias was low-medium.

**Conclusion:**

In conclusion, this review indicated the critical role of the *IRF6* gene and its downstream genes (*GRHL3, KLF17,* and *ESRP1/2*) in the development of cleft lip and palate in zebrafish models. Genetic mutation zebrafish models provide a high level of insights into zebrafish craniofacial development.

**Clinical relevance:**

this review provides a productive avenue for understanding the powerful and conserved zebrafish model for investigating the pathogenesis of human cleft lip and palate.

## Highlights

• The study focuses on the crucial role of *IRF6* in palatal development.
• Zebrafish are a powerful tool for studying human craniofacial development.
• Zebrafish advantages include transparency, rapid development, and affordability.
• *IRF6* and its downstream genes are essential in zebrafish palatogenesis.
• Studies on zebrafish dentition may offer new findings in the dental field.

## 1 Introduction

Understanding craniofacial growth and development is essential for clinicians to improve the diagnosis and treatment of patients with dentofacial deformities and craniofacial anomalies, such as cleft lip and palate and craniosynostosis [1]. Craniofacial development is initiated by the ventrolateral migration of cranial neural crest cells (CNCCs) from the dorsal neural tube to create the ectomesenchyme of the frontonasal prominence and pharyngeal arches [2,3]. Genetic and environmental factors significantly influence craniofacial growth and development [4]. Identifying the genes controlling craniofacial development is vital to understanding the genetic etiology of craniofacial abnormalities, such as cleft lip and palate and craniosynostosis [5].

Cleft lip and palate are the most common craniofacial birth anomaly [6]. They can be associated with syndromes or occur separately [6]. The global prevalence of cleft lip and palate is 0.45 in every 1000 live births [7]. Cleft lip and palate affect the quality of life and lead to severe speech, nutrition, and social or mental developmental abnormalities [8]. Several genes and genetic pathways have been implicated in human cleft pathogenesis [9]. The interferon regulatory factor 6 (*IRF6)* is a key transcriptional regulator of palatal development [10,11]. Mutations in *IRF6* contribute to cleft lip and palate [12]. Like their mammalian counterparts, zebrafish deficient in *IRF6* demonstrate a cleft phenotype [13,14]. Therefore, the conserved genetic network regulating the face across vertebrates supports using zebrafish as a model for the mammalian hard palate [15].

**Fig. 1:**
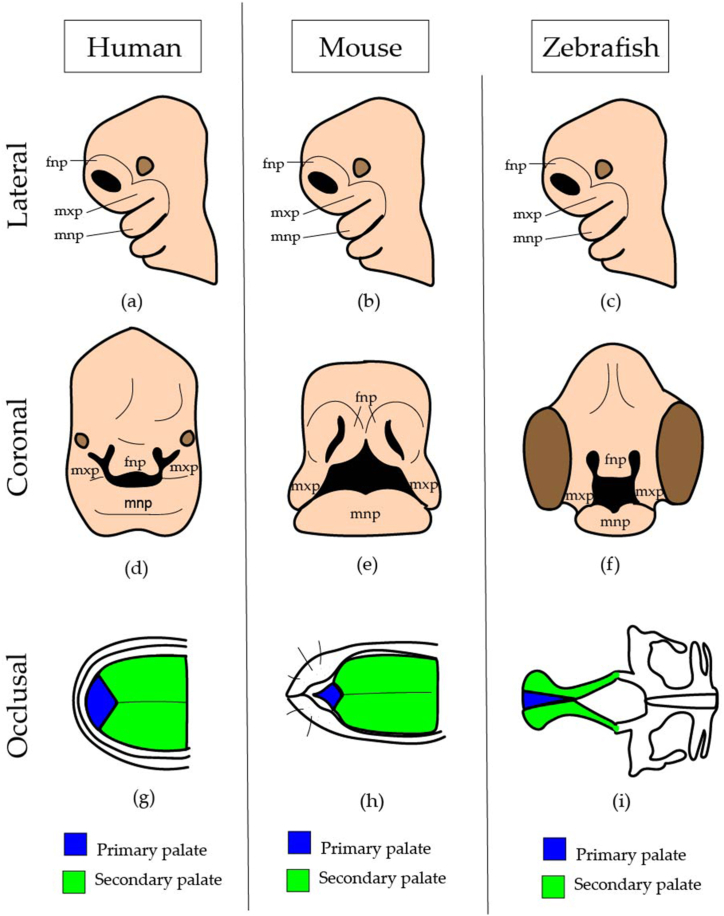
This is a figure illustrating the conserved pharyngeal arch patterning across vertebrates. (a),(b) and (c) demonstrate lateral view showing early pharyngeal arch patterning is conserved across vertebrates; (d), (e), and (f) show coronal cross-sectional view demonstrating the facial development of human, mouse, and zebrafish embryos. (g), (h) and (i) illustrating the zebrafish model of the mammalian hard palate. The primary palate (blue color) and secondary palate (green color) in humans, mice, and zebrafish. Abbreviations: fnp: frontonasal prominence, mxp: maxillary prominence, mnp: mandibular prominence. (For interpretation of the references to color in this figure legend, the reader is referred to the Web version of this article.)

Early pharyngeal arch patterning is conserved across vertebrates, supporting the use of zebrafish models to understand human craniofacial development (Fig. 1a–f) [16]. The zebrafish system has the advantages of transparent embryos and rapid external development, which allows visualization of all stages of development and is suitable for high-resolution imaging [15,17]. In addition, it is possible to visualize changes in gene expression and detailed morphology in zebrafish using fluorescent reporter genes, which were first described by Stuart et al. (1988) [17,18]. Furthermore, forward and reverse genetics, tissue-specific clustered regularly interspaced palindromic repeats (CRISPR)/CRISPR-associated nuclease (Cas) gene editing techniques, and affordability are the advantages of zebrafish models [19]. Moreover, the major contribution of the zebrafish model to vertebrate development is the possibility of real-time imaging of developing embryos [17]. Interestingly, there are unique conserved structures in humans and zebrafish, such as the fusion of the frontonasal and maxillary neural crests to form the mammalian hard palate and the zebrafish ethmoid plate (functionally equivalent to the mammalian palate) [13]. In zebrafish, the ethmoid plate originates from the cranial neural crest to form the roof of the mouth/cranial base. It is derived from the convergence of three distinct parts: the frontonasal median element derived from the most anterior stream of migrating CNCCs and paired maxillary elements derived from the second stream of CNCCs [14,20]. Thus, the zebrafish ethmoid plate (palate) is analogous to the mammalian palate (Fig. 1g–i) [21].

Although experimental studies have been carried out to understand the role of the *IRF6* gene and its downstream targets in cleft lip and palate pathogenesis, major gaps in our knowledge remain in drafting a comprehensive overview of the contribution of the *IRF6* gene to the formation of cleft lip and palate in the zebrafish model. Therefore, this systematic review provides evidence supporting the role of the *IRF6* gene and its downstream targets in cleft lip and palate etiology in a zebrafish model. In this review, we focused on the zebrafish system because it is a powerful and conserved model for studying human cleft lip and palate pathogenesis. This study provides novel insights into phenotypes associated with *IRF6* gene mutations during craniofacial development in zebrafish for craniofacial researchers.

## 2 Materials and methods

This review adhered to the guidelines outlined in the Preferred Reporting Items for Systematic Reviews and Meta-Analyses (PRISMA) statement [22]. The systematic review protocol was registered in the International Prospective Register of Systematic Reviews (PROSPERO) (registration number CRD42023464763).

The research question was formulated using the participant, intervention, comparison, and outcome (PICO) models. This study aimed to investigate the influence of the *IRF6* gene and its downstream genes (*GRHL3, KLFL17,* and *ESRP1/2*) on the development of cleft lip and palate in zebrafish models. Specifically, this study focused on all zebrafish models with experiments related to cleft lip and palate and mutations in *IRF6* and its downstream genes, compared to controls lacking mutations in these genes. The primary outcome of interest was the presence or absence of cleft lip and palate phenotypes.

We conducted a systematic search of the electronic bibliographic databases MEDLINE, EMBASE, and Web of Science, covering the period from their inception until September 15, 2023. In addition, we performed a comprehensive search of the Zebrafish Information Network (ZFIN), a dedicated database of the zebrafish model organism [23]. Our search encompassed experimental studies utilizing a zebrafish model (*Danio rerio*) to investigate cleft lip and palate formation. Reviews, abstracts, theses, dissertations, and conference abstracts were excluded. The search strategy is shown in (Table 1). Non-English articles were considered if the English-translated version was available in the respective journal. Furthermore, the reference lists of the selected studies were manually examined to identify additional relevant studies. We attempted to contact authors with missing data.

**Table 1:** Search strategy for the databases.

Database	Search terms	Total
MEDLINE	(“Zebrafish"[MeSH Terms] OR “zebrafish∗"[All Fields] OR “Danio rerio"[All Fields]) AND (“*irf6* protein zebrafish"[Supplementary Concept] OR “Cleft Palate"[MeSH Terms] OR “Cleft Lip"[MeSH Terms] OR “Ethmoid Bone"[MeSH Terms] OR “ethmoid plate"[All Fields] OR “Cleft Lip"[All Fields] OR “Cleft Palate"[All Fields] OR (“cleft"[All Fields] OR “clefted"[All Fields] OR “clefting"[All Fields] OR “clefts"[All Fields]) OR “*irf6*"[All Fields])	191
EMBASE	#1 Zebrafish.mp. or exp zebra fish/#2 Danio rerio.mp. or zebra fish/#3 Ethmoid Bone.mp. or exp ethmoid bone/#4 Cleft Lip.mp. or exp cleft lip/#5 Cleft Palate.mp. or exp cleft palate/#6 ethmoid∗ plate.mp. #7 cleft.mp. #8 irf6.mp. or exp interferon regulatory factor 6/#9 #3 or #4 or #5 or #6 or #7 or #8 #10 #1 or #2 #11 #9 and #10	267
Web of Science	((Zebrafish∗ OR “Danio rerio”) AND (“ethmoid∗ plate” OR “cleft lip” OR “cleft palate” OR cleft OR *irf6*))	818

We used a systematic review management software tool (COVIDENCE™) (version 2.0; Covidence, Melbourne, Australia) for screening and data extraction. The screening process involved independent evaluation of titles and abstracts by two authors, N.A. and K.A.A. If both authors agreed that a study did not meet the inclusion criteria, it was excluded. Any discrepancies at this stage were resolved through consultation with the third author, F. A. Subsequently, the same two reviewers, N.A. and K.A.A., independently assessed the full texts of the papers that met the initial screening criteria. Any disparities at this stage were resolved by a third author, F. A., to reach a final consensus.

All selected studies were subjected to risk of bias assessment, conducted independently by N.A. and K.A.A., utilizing the Systematic Review Center for Laboratory Animal Experimentation (SYRCLE)'s risk of bias tool [24]. A third reviewer, F.A., resolved any disagreements during this assessment.

For the synthesis of the findings, a narrative approach was employed, organized around key factors, including zebrafish strain, age, study aims, assessed variables (*IRF6* or its downstream genes), gene editing techniques, observed phenotypes, and the main results of the study. The outcomes related to these factors and their respective references are summarized (Table 2).

**Table 2:** Characteristics of included studies. Abbreviations**:** CRISPR: Clustered regularly interspaced short palindromic repeats, dpf: days post-fertilization, *esrp*: epithelial splicing regulatory protein, gfp: green fluorescent protein, *ghl*: grainy head-like transcription factor, zGPAEs: zebrafish green fluorescent protein, positive active enhancers, hpf: hours post-fertilization, *irf6:* Interferon regulatory factor 6, *KLF*: Krüppel-like factor, KRT4: keratin 4, tg: transgenic, MO: morpholino. NHGRI: National Human Genome Research Institute, NM: mRNA, *wnt9a*: Wnt family member 9a.

Authors	Year	Zebrafish strain	Zebrafish age	Study aim	Assessed variable	Gene editing	Phenotype	Main results
Sabel et al. [34]	2009	Not mentioned	4, 4.3, 6, 7, 8.3, 8.5, 13, 20, 22, 24 hpf 4 dpf	To investigate the requirement of maternal *IRF6* in the differentiation of primary superficial epithelium during development.	Maternal *IRF6*	MO	Embryonic rupture	- *IRF6* is expressed in cleavage-stage embryos, confirming the presence of maternal *IRF6*. - Dominant negative *IRF6* disrupts gastrulation. - *IRF6* is required for the epiboly of the three cell layers.
De La Garza et al. [11]	2013	tg(krt4:gfp) transgenic	6, 6.5, 7, 7.5, 11, 24, 48, and 72 hpf	To identify genes that are expressed in the zebrafish periderm and whose expression is inhibited by a dominant-negative variant of *IRF6.*	*GRHL1, GRHL3*	CRISPR	Embryonic rupture	- The study supports that *IRF6* directly activates *GRHL3* expression in human keratinocytes and the zebrafish periderm.
Dougherty et al. [14]	2013	Not mentioned	16, 19, 24, 48, 55 60, 72 hpf 4.5 dpf	To present a detailed analysis of zebrafish palatogenesis, demonstrating that *WNT9A* and *IRF6* are required for distinct morphogenetic processes.	*WNT9A, IRF6*	MO	short ethmoid plate, clefts, dysmorphic lower jaw	*- IRF6* is required specifically to integrate facial prominences along a V-shaped seam.
Liu et al. [32]	2016	NM_131723.1	5, 6, 24, and 72 hpf	To identify candidate genes for cleft lip and palate pathogenesis.	*KLF17*	CRISPR	Embryonic rupture	- *KLF17* acts downstream of *IRF6* influencing zebrafish periderm differentiation. -*IRF6* directly regulates *KLF17* expression in zebrafish periderm.
Leslie et al. [33]	2016	NHGRI line	6, and 8 hpf	To identify the risk factors for non-syndromic cleft palate.	*GRHL3*	MO	Embryonic rupture	- There is an association between a missense variant (rs41268753) *GRHL3* and cleft palate
Li et al. [29]	2017	Tübingen strain	4, 24, and 96 hpf	To test the protein functions of human *IRF6* missense gene variants and provide an additional line of biological evidence to help bridge the gap between gene variant identification and pathogenicity assignment.	*IRF6*	CRISPR	Embryonic rupture	- Ablation of *IRF6* function during early embryogenesis significantly disrupts the expression of genes critical for epithelial and craniofacial morphogenesis that can be rescued by zebrafish or human *IRF.*
Liu et al. [31]	2020	Tg(krt4:gfp) transgenic	4, 5.25, 6, 8, 10, 11, 15, 24, 52 hpf 5 dpf	To learn more about the gene regulatory network governing periderm differentiation using zebrafish periderm enhancers and training a classifier with which to prioritize SNPs in non-coding DNA for their likelihood of disrupting periderm enhancers.	*IRF6, GRHL3,* and *KFL17*	CRISPR	Cleft lip and palate	- Most or perhaps all zGPAEs are enhancers active in periderm in embryos at 8 hpf to 24 hpf. -There is modest but detectable sequence homology between human and zebrafish.
Carroll et al. [30]	2020	Tübingen strain	3, 4, 5, 10, 12, 15, 10, 48, 72, 96, 120 hpf 4, 5, 9 dpf	To provide detailed gene expression analysis of *IRF6, ESRP1,* and *ESRP2* in zebrafish and to understand the facial morphogenesis. Also, to compare *IRF6, ESRP1/2* mutant phenotypes	*IRF6* and *ESRP1/2*	CRISPR MO	Cleft lip and palate Short palate	*- IRF6* null zebrafish have reduced *ESRP1* expression. - Co-expression of *IRF6*, *ESRP1* and *ESRP2* in oral epithelium during development. -The compound mutation of *ESRP1* and *ESRP2* results in cleft lip and palate. - *IRF6* and *ESRP1/2* function in the same pathway.
Huang et al. [35]	2023	Not mentioned	Not mentioned	To identify a rare *GRHL3* variant underlying non-syndromic cleft palate only among Chinese patients.	*GRHL3* rare variant *GRHL3* p.Arg391His)	Inject human gene variant in zebrafish embryo	Rupture Autonomous movement defects	- *GRHL3* p.Arg391His variant disrupts the normal development of zebrafish embryos. - *GRHL3* p.Arg391His variant altered the *GRHL3* transcriptional activity.

## 3 Results

In the present study, two reviewers (N.A. and K.A.A.) performed the screening process in 20% increments until all eligible records were identified. According to Cohen's kappa coefficient [25], the inter-rater reliability of the data screening indicates a moderate agreement level (values ranging between 0.69 and 0.74).

**Fig. 2:**
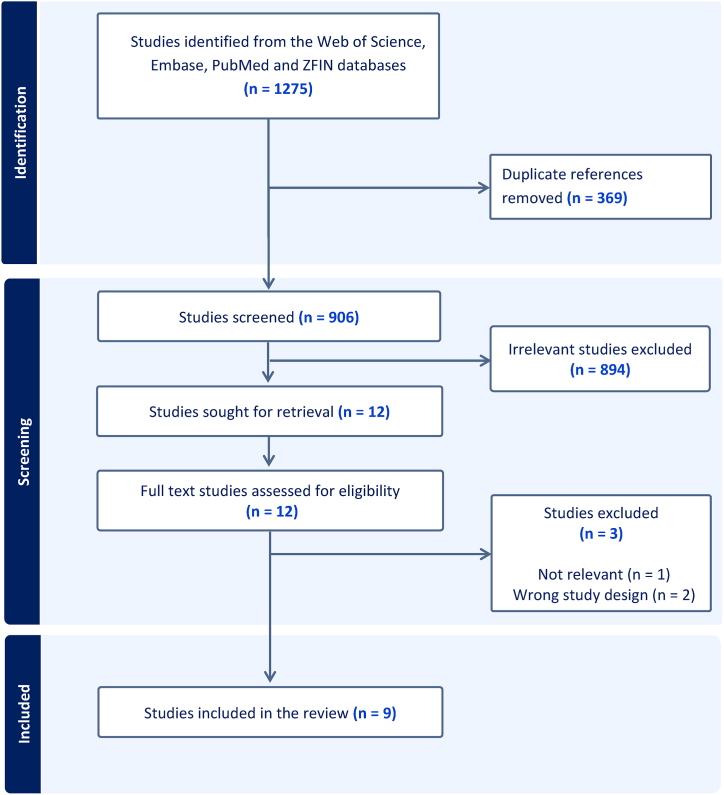
PRISMA flowchart of studies selected in the review.

This systematic review identified 1275 articles, of which 369 duplicates were eliminated using COVIDENCE™. After screening titles and abstracts, 894 irrelevant articles were excluded, making 12 articles eligible for full-text screening. During the full-text screening, we excluded three additional studies [[26], [27], [28]] because of wrong study design and not relevant to our study aim, resulting in nine studies being included in this systematic review. The PRISMA flow diagram summarizes the screening process (Fig. 2). The selected studies’ characteristics are described (Table 2).

**Fig. 3:**
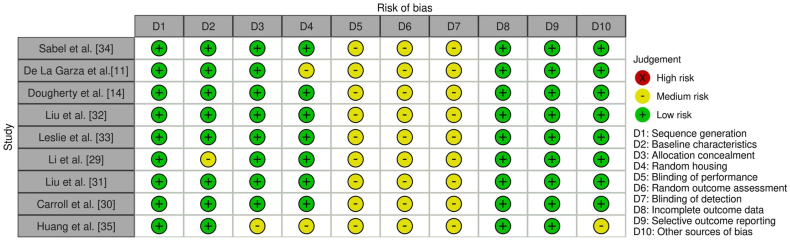
Individual SYRCLE's risk of bias assessment. Abbreviation: SYRCLE: Systematic Review Centre for Laboratory Animal Experimentation.

Two studies used similar zebrafish strains (Tübingen strain) [29,30], and two other studies used Tg(krt4:gfp) [11,31]. Zebrafish ages in the included studies ranged from 4 h post-fertilization (hpf) to 9 days post-fertilization (dpf). Regarding genetic editing tools, some studies have used CRISPR [11,29,32,33]; in contrast, others have used morpholino antisense oligonucleotides [14,31,33,34]. However, Carroll et al. [30] used CRISPR and morpholino techniques, and Huang et al. [35] injected human gene variants into zebrafish embryos. The individual SYRCLE risk of bias assessment ranged between low and moderate, as demonstrated (Fig. 3).

## 4 Discussion

Cleft lip and palate are the most common congenital disabilities [6]. Insights into the genetic factors that contribute to cleft lip and palate pathogenesis are important for clinicians to achieve proper diagnosis and treatment planning. Studies have identified the *IRF6* gene as a key transcription factor associated with the pathogenesis of cleft lip and palate pathogenesis [12,36,37]. To our knowledge, this is the first systematic review to unravel the role of the *IRF6* gene and its downstream genes (*GRHL3, KLF17,* and *ESRP1/2*) in developing cleft lip and palate in zebrafish models.

The main outcome of the included studies supports the role of the *IRF6* gene in zebrafish periderm development, embryogenesis, and gastrulation and that variations in *IRF6* result in cleft lip and palate development. *IRF6* null zebrafish resulted in embryonic rupture and cleft lip and palate [14,29,31,34]. In addition, disruption of the *IRF6* downstream targets*, GRHL3, KLF17,* and *ESPR1/2* demonstrated embryonic rupture, cleft lip and palate, short palate, and autonomous movement defects, indicating the importance of *IRF6* and its downstream genes in palatal development and embryogenesis [11,30,32,33,35].

We focused on the *IRF6* transcription factor because it is one of the most commonly associated genes with cleft lip and palate, based on recent genome-wide association studies and studies carried out over a decade [[37], [38], [39], [40]]. Several *IRF6* transcriptional targets have been identified, such as *GRHL3, KLF17,* and *ESRP1/2* [11,30,32]. Because genetics play a major role in cleft lip and palate pathogenesis, environmental factors were excluded. Moreover, as this study included a zebrafish (Danio rerio) experimental model, the ZFIN database was used. This online database provides a wide range of genomic data and integrated analyses of genotype, phenotype, and expression information for zebrafish models and future trends to alter gene function [23].

There is a debate in the zebrafish community regarding gene knockdown versus knockout techniques that has been ongoing for over a decade [41]. Genome editing tools allow the silencing of target gene function to analyze the phenotype and identify the gene's function of interest [42]. The knockout approach refers to the ablation of gene function; in contrast, the knockdown approach refers to the repression of gene expression [42]. Some of the included studies utilized a gene knockout approach using CRISPR Cas 9 [11,29,31,32]; in contrast, other studies applied gene knockdown using morpholino-modified oligonucleotides [14,30,33,34]. In addition, one study induced gene knockdown by injecting the mRNAs of the detected human mutant *GRHL3* into zebrafish embryos [35]. In the De La Garza et al. [11] study, the authors justified using CRISPR Cas 9 instead of antisense morpholino oligonucleotides because it is commonly effective against maternally encoded transcripts. Although morpholinos can exert non-specific off-target effects irrelevant to target gene function, they do not display transcriptional adaptation and target maternal and zygotic mRNA, unlike knockout tools [42]. Therefore, the use of morpholino knockdown techniques along with knockout experiments is still advocated [42]. However, it is crucial to consider controlling the morpholino dose to control off-target effects [42]. We suggest using knockout and knockdown techniques to gain a better understanding of the target gene function.

In this study, we sought to highlight the strengths of zebrafish as a model for studying the development of human cleft lip and palate. The included studies showed that *IRF6* and its downstream genes (*GRHL3, KLF17,* and *ESRP1/2*) were expressed in the developing palate of zebrafish. We also showed that zebrafish genes could be modulated by different loss-of-function experiments, which improved our understanding of human gene functions. Additionally, we observed a high degree of genetic similarity between humans and zebrafish. Moreover, experimental analysis of zebrafish embryos allowed us to identify *IRF6* transcription factor binding sites, which improved our understanding of *IRF6* regulation in wild-type and mutant *IRF6* [11,14,[29], [30], [31], [32], [33], [34]].

The overall SYRCLE risk of bias in the included studies was low to medium. However, it is essential to mention that based on the study design of the included paper, some domains were not applicable, such as sequence generation and allocation concealment. Therefore, these items were assessed as having a low risk of bias. Hence, the assessment of animal quality requires modifications and improvements to assess the actual risk of bias [43]. In addition, many details in the conduct of zebrafish experiments, such as blinding of zebrafish groups or experimental outcome assessment, were often unclear (whether these measures were applied or not) but not reported. Moreover, details regarding the zebrafish strain, maintenance, sample size, sample replicates, negative controls (embryos injected with standard control oligonucelotides), and positive controls (embryos without any injection) were not reported. Understanding the findings of the included studies requires careful consideration of several limitations. These include the absence of information regarding the specific zebrafish strains because different strains may exhibit distinct genetic backgrounds. Furthermore, the reliance on diverse gene editing techniques introduces methodological heterogeneity, potentially influencing the reproducibility and comparability of results. In addition, the absence of uniformity in reporting experimental controls and details, such as zebrafish maintenance, sample size, and replicates, may also limit the robustness of the conclusions drawn from these studies [11,14,[29], [30], [31], [32], [33], [34]]. Therefore, it is recommended that scientists embrace the use of blinding in animal allocation and outcome assessment and provide detailed information regarding experimental conduction to improve the methodological quality of the study.

**Fig. 4:**
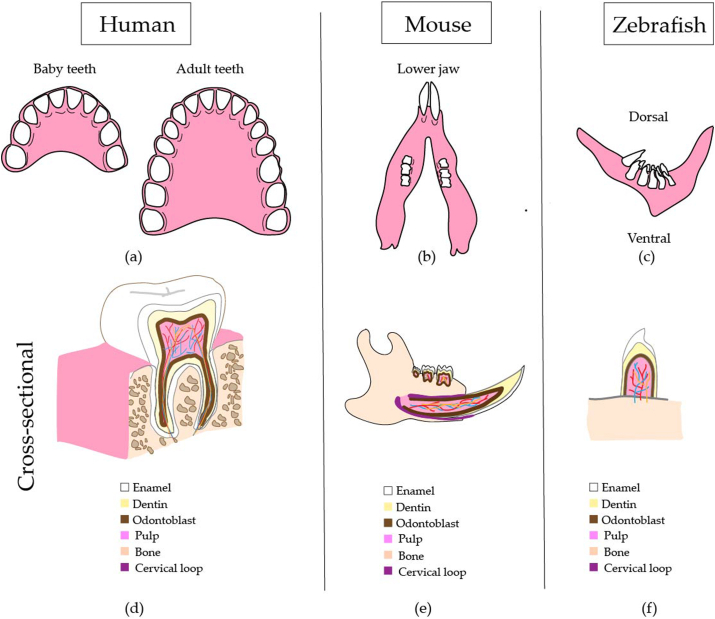
This figure shows analogous dental anatomy in humans, mice, and zebrafish. (**a**) Two sets of human teeth; (**b**) Mouse lower jaw with a single set of three molars and an incisor on each side; **(c)** Zebrafish jaw with five teeth in the ventral row, four teeth in the mid-dorsal row and two teeth in the most dorsal row; **(d)** Schematic cross-section of lower molar showing dental layers; **(e)** Unlike human teeth, mice have continuously growing lower incisors from stem cells in the cervical loop; (**f**) Zebrafish teeth continuously replaces themselves. However, zebrafish teeth do not have roots, and the tooth's crown is ankylosed to the bone.

Taken together, our systematic review indicated the critical role of the *IRF6* gene and its downstream genes (*GRHL3, KLF17,* and *ESRP1/2*) in the development of cleft lip and palate in zebrafish models. Genetic mutation zebrafish models provide a high level of insights into zebrafish craniofacial development. Therefore, utilization of the zebrafish model to study the development of genetic mechanisms involved in craniofacial abnormalities is recommended for future studies. Because dental anomalies are highly associated with cleft lip and palate, well-designed studies on zebrafish dentition may offer new findings in the dental field [44,45]. It has been reported that zebrafish tooth structures are similar to mammalian teeth, which justifies their use to study human dental abnormalities (Fig. 4a–f) [46]. Fig. 4a–f shows the similarities in the dental structure between humans, mice, and zebrafish.

## 5 Conclusions

In this review, we have highlighted the conserved early pharyngeal arch patterning across vertebrates, which values the zebrafish models used to understand human craniofacial development. In addition, zebrafish share some features with the human hard palate. The main outcome of this study highlights the role of the *IRF6* gene in zebrafish in palatogenesis and disruption of *IRF6* or its downstream targets*; GRHL3, KLF17,* and *ESPR1/2* demonstrated cleft lip and palate development, indicating the importance of *IRF6* and its downstream genes in palatal development and embryogenesis. Despite the differences between humans and zebrafish, we demonstrated that zebrafish are a powerful tool for studying human craniofacial development.

## Funding statement

This research was funded by the 10.13039/501100013302King Abdullah International Medical Research Center (KAIMRC; grant number NRC23R/610/10).

## Data availability statement

Data included in article/supplementary Material/referenced in article.

**ORCID:** Nora Alhazmi https://orcid.org/0000-0002-3873-4910.

## CRediT authorship contribution statement

**Nora Alhazmi:** Writing – review & editing, Writing – original draft, Validation, Resources, Project administration, Investigation, Funding acquisition, Formal analysis, Conceptualization. **Khalid A. Abalkhail:** Writing – review & editing, Validation, Investigation, Formal analysis, Data curation, Conceptualization. **Farraj Albalawi:** Writing – review & editing, Validation, Investigation, Formal analysis, Data curation, Conceptualization. **Bassam Alalola:** Writing – review & editing, Writing – original draft, Validation, Software, Methodology. **Fathima F. Farook:** Writing – original draft, Validation, Methodology.

## Declaration of competing interest

The authors declare that they have no known competing financial interests or personal relationships that could have appeared to influence the work reported in this paper.
